# Supplementary material for: Low efficacy of recombinant SV40 in Ugt1a1-/- mice with severe inherited hyperbilirubinemia
Source: PLoS One. 2021 Apr 23;16(4):e0250605. doi: 10.1371/journal.pone.0250605 (PMC8064607; doi:10.1371/journal.pone.0250605)
Supplement: S1 Fig — 60-day-old Ugt1a-/- mice were treated with 2x10^11^ vg/kg of rSV-hUGT1A1 vector (n = 3), or rSV-HLP-hUGT1A1 (n = 3) via tail vein injection. At 8 weeks after vector administration the level of anti-hUGT1A1 IgG in serum was determined. Data represent the mean ± SD. (PDF) [file pone.0250605.s001.pdf]

S1 Fig

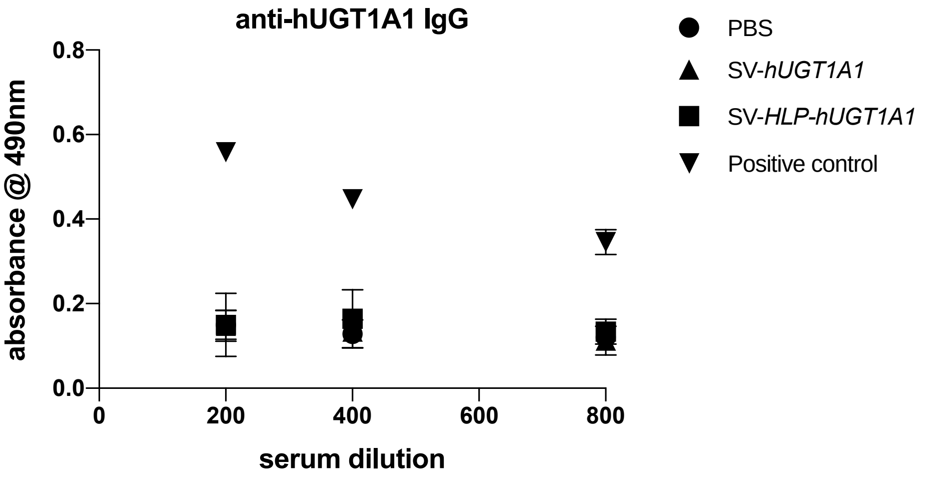

S1 Fig. Absence of antibodies towards hUGT1A1 in serum of mice treated with rSV-h*UGT1A1* or rSV-HLP-h*UGT1A1*. 60-day-old Ugt1a $^{-/-}$ mice were treated with $2x10^{11}$ vg/kg of rSV-h*UGT1A1* vector (n=3), or rSV-HLP-h*UGT1A1*(n=3) via tail vein injection. At 8 weeks after vector administration the level of anti-hUGT1A1 IgG in serum was determined. Data represent the mean ± SD.
