# Supplementary material for: Low efficacy of recombinant SV40 in Ugt1a1-/- mice with severe inherited hyperbilirubinemia
Source: PLoS One. 2021 Apr 23;16(4):e0250605. doi: 10.1371/journal.pone.0250605 (PMC8064607; doi:10.1371/journal.pone.0250605)
Supplement: S1 Raw images — (PDF) [file pone.0250605.s002.pdf]

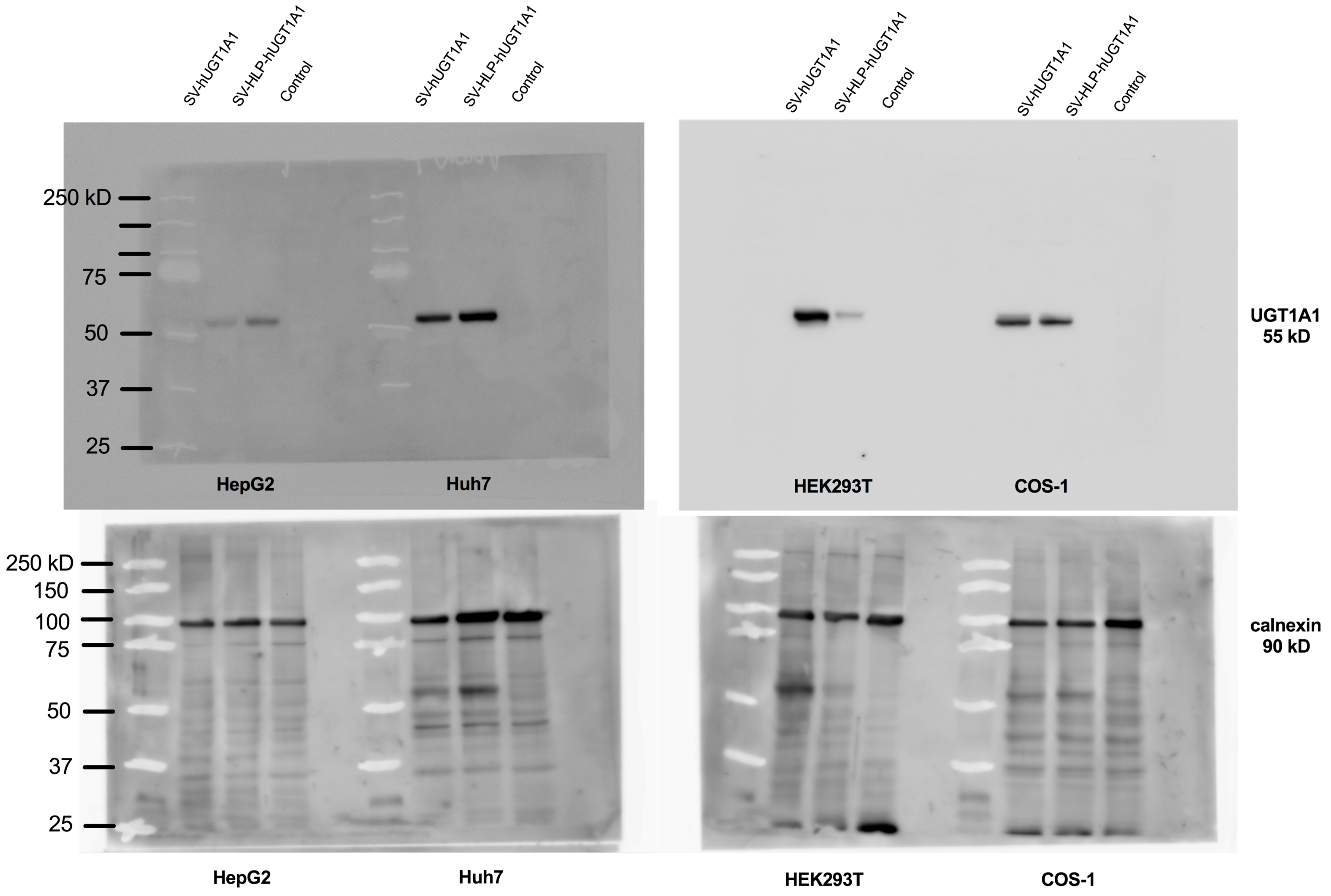

Figure 3 panel A were generate from these original blots

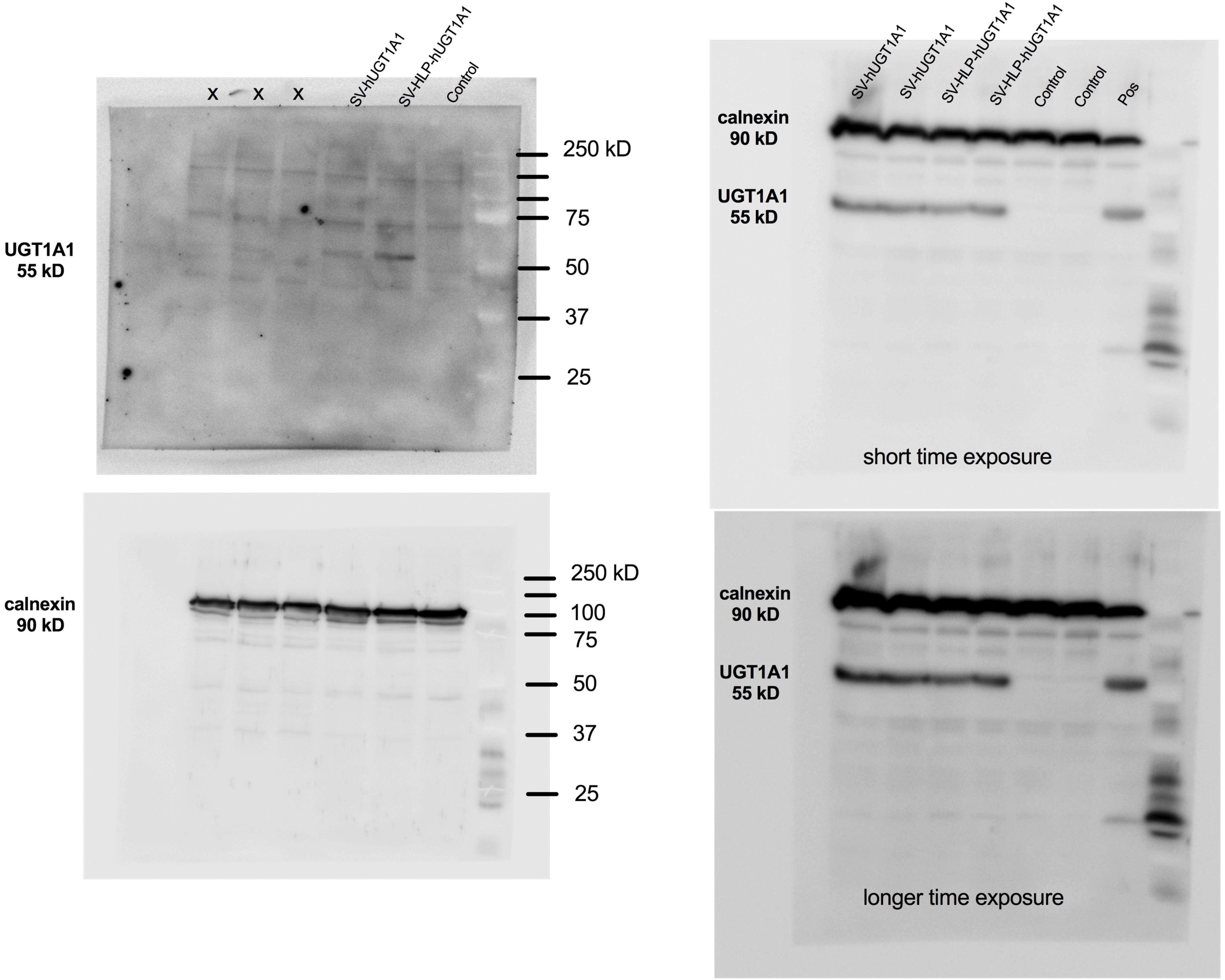

Figure 3 panel C were generate from these original blots

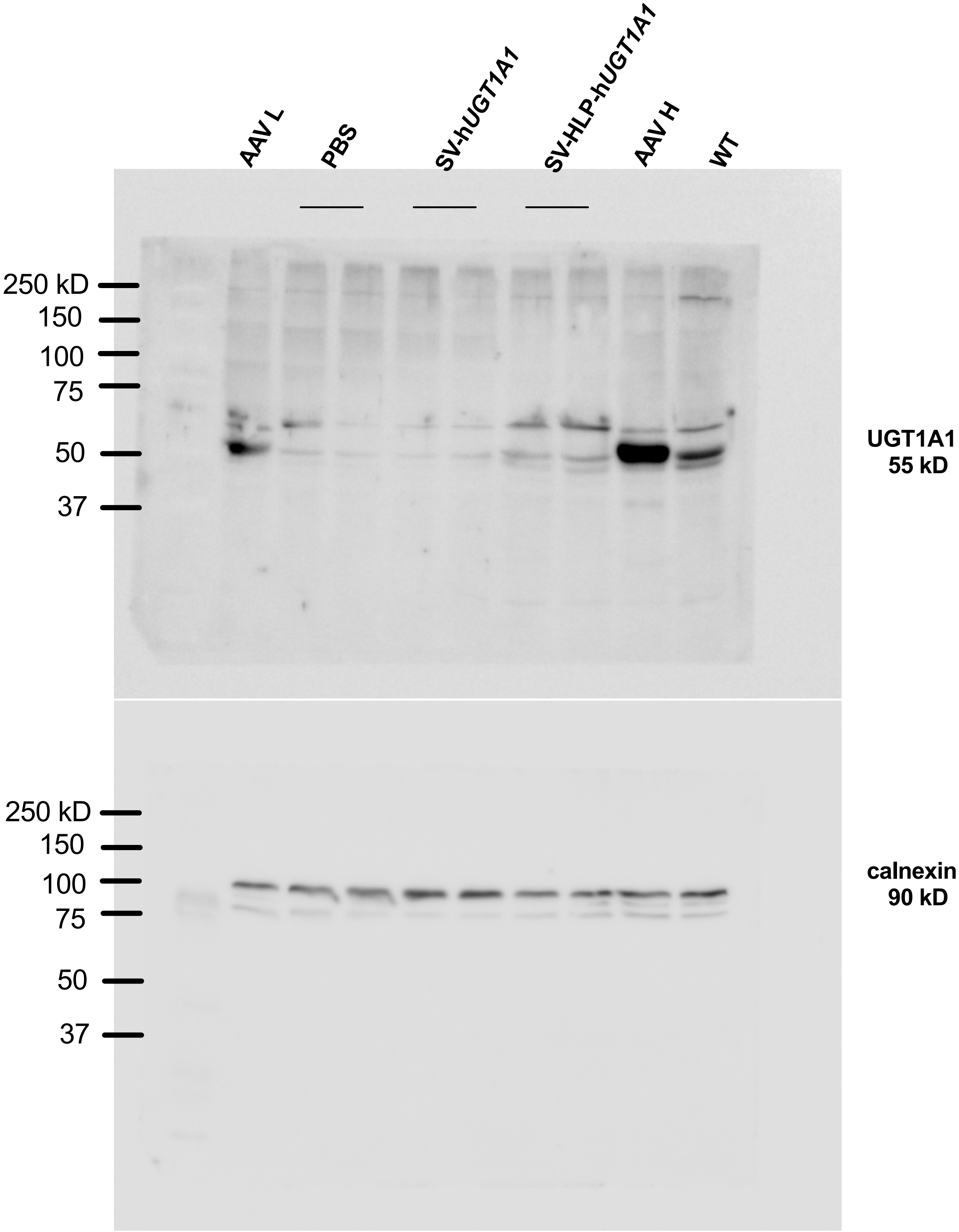

Figure 4 panel D were generate from these original blots
