# Supplementary material for: Low efficacy of recombinant SV40 in Ugt1a1-/- mice with severe inherited hyperbilirubinemia
Source: PLoS One. 2021 Apr 23;16(4):e0250605. doi: 10.1371/journal.pone.0250605 (PMC8064607; doi:10.1371/journal.pone.0250605)
Supplement: Attachment — Submitted filename: Review-PONE-D-20-33243.docx [file pone.0250605.s003.docx]

This paper describes simian virus 40 (rSV40) is difficult to serve as candidate vector for *in vivo* gene therapy, due inefficient production and induction of immune neutralization. The study provides a novel sight that rSV40 triggered neutralizing antibody production, inconsistent with previous reports. Most of results are solid and convincing. However, some concerns and more experiments are need to further verify.

1. *In vitro*, authors used the human liver-derived cell lines (Huh7 and HepG2) to test the activation of hybrid liver specific promoter, they should use mouse liver-derived cell lines to test the promoter activity as well.

2. Authors are necessary to detect the expression of hUGT1A1 in mRNA and protein levels in liver from mice injected by rSV40 vectors.

3. In figure 4 and 5, statistical analysis methods should be included in figure legends.

4. There is no data description the “lack of purification method to produce pure high tittered batches” in title, hence the title needs to be amended.

5. Authors needs to present some data about the *Ugt1a* gene being knocked out in mice.

6. English/grammar should be improved for clarity, e.g. in 173-174 line, wass should be corrected to was.
