# Supplementary material for: Low efficacy of recombinant SV40 in Ugt1a1-/- mice with severe inherited hyperbilirubinemia
Source: PLoS One. 2021 Apr 23;16(4):e0250605. doi: 10.1371/journal.pone.0250605 (PMC8064607; doi:10.1371/journal.pone.0250605)
Supplement: Attachment — Submitted filename: Response to reviewers.docx [file pone.0250605.s004.docx]

Dr. P. J. Bosma

Amsterdam University Medical Centers

Tytgat Institute for Liver Research

Meibergdreef 69

1105 BK Amsterdam

The Netherlands

Dr. Joerg Heber

Editor-in-Chief

PLOS ONE

February 1st 2021

Dear dr. Joerg Heber,

We are grateful for your potential interest in our manuscript entitled manuscript “*Lack of purification method to produce pure high tittered batches, renders rSV40 a poor candidate for in vivo liver directed gene therapy* (ID: PONE-D-20-33243)”. We thank the reviewers for their comments and suggestions to improve our manuscript. A manuscript with the tracked changes in red and a clean version are submitted. Please find our point-by-point response below.

Yours sincerely,

Xiaoxia Shi

Dr. Piter Bosma

**Journal Requirements:**

1. Please ensure that your manuscript meets PLOS ONE's style requirements, including those for file naming. The PLOS ONE style templates can be found at

https://journals.plos.org/plosone/s/file?id=wjVg/PLOSOne_formatting_sample_main_body.pdf and

https://journals.plos.org/plosone/s/file?id=ba62/PLOSOne_formatting_sample_title_authors_affiliations.pdf

2. Thank you for including your ethics statement:  "Mice were maintained in the International Centre for Genetic Engineering and Biotechnology animal house unit. All animal studies were approved by the local ethical committee. ".

*Please amend your current ethics statement to include the full name of the ethics committee that approved your specific study.*

For additional information about PLOS ONE submissions requirements for ethics oversight of animal work, please refer to http://journals.plos.org/plosone/s/submission-guidelines#loc-animal-research

Once you have amended this/these statement(s) in the Methods section of the manuscript, please add the same text to the “Ethics Statement” field of the submission form (via “Edit Submission”).

**Response:** we have amended the ethics statement according to the requirement in the manuscript.

Line 122-124: All animal protocols were approved by the Animal Welfare and Ethics Committee of the International Centre for Genetic Engineering and Biotechnology (ICGEB), Trieste, Italy.

3. In your Methods section, please provide additional information on the animal research and ensure you have included details on: (1) methods of sacrifice (2) methods of anesthesia and/or analgesia, and (2) efforts to alleviate suffering.

**Response:** According to the requirements, we have added the details in the manuscript.

Line 127-134: Intravenous of rSV40 was performed under isoflurane anesthesia. No surgical or other procedures requiring anesthesia were performed. Animals were treated by researchers trained for animal care and monitored daily for general appearance. No signs of stress (lack of appetite, body weight loss, hair loss, stereotypic or aggressive behavior, etc.) or macroscopic alterations of vital functions were observed throughout the experimental phase. Blood was sampled at 1, 3, 5 and 8 weeks after vector administration by facial vein puncture and collected in EDTA-containing tubes. At the time of sacrifice mice were anesthetized with 5% isoflurane, cardiac puncture was performed to obtain blood samples, and sacrificed by cervical dislocation.

4. PLOS ONE now requires that authors provide the original uncropped and unadjusted images underlying all blot or gel results reported in a submission’s figures or Supporting Information files. This policy and the journal’s other requirements for blot/gel reporting and figure preparation are described in detail at https://journals.plos.org/plosone/s/figures#loc-blot-and-gel-reporting-requirements and https://journals.plos.org/plosone/s/figures#loc-preparing-figures-from-image-files. When you submit your revised manuscript, please ensure that your figures adhere fully to these guidelines and provide the original underlying images for all blot or gel data reported in your submission. See the following link for instructions on providing the original image data: https://journals.plos.org/plosone/s/figures#loc-original-images-for-blots-and-gels.

In your cover letter, please note whether your blot/gel image data are in Supporting Information or posted at a public data repository, provide the repository URL if relevant, and provide specific details as to which raw blot/gel images, if any, are not available. Email us at plosone@plos.org if you have any questions.

**Response:** We have provided the original underlying images for all blot reported in our manuscript with the name of S1_raw_images.

5. Thank you for stating the following in the Acknowledgments Section of your manuscript:

"Xiaoxia Shi is recipient of a fellowship of the China Scholarship Council (CSC)."

We note that you have provided funding information that is not currently declared in your Funding Statement. However, funding information should not appear in the Acknowledgments section or other areas of your manuscript. We will only publish funding information present in the Funding Statement section of the online submission form.

Please remove any funding-related text from the manuscript and let us know how you would like to update your Funding Statement. Currently, your Funding Statement reads as follows:

"The author(s) received no specific funding for this work."

Please include your amended statements within your cover letter; we will change the online submission form on your behalf.

**Response:** We have deleted the sentence ‘Xiaoxia Shi is recipient of a fellowship of the China Scholarship Council (CSC)’ in the Acknowledgments section and want to include this in the Funding Statement.

6. We note that you have included the phrase “data not shown” in your manuscript. Unfortunately, this does not meet our data sharing requirements. PLOS does not permit references to inaccessible data. We require that authors provide all relevant data within the paper, Supporting Information files, or in an acceptable, public repository. Please add a citation to support this phrase or upload the data that corresponds with these findings to a stable repository (such as Figshare or Dryad) and provide and URLs, DOIs, or accession numbers that may be used to access these data. Or, if the data are not a core part of the research being presented in your study, we ask that you remove the phrase that refers to these data.

**Response:** we have added the results as supplemental figure with the name of S1 Fig.

**Reviewers' comments:**

**Reviewer #1:** This paper describes simian virus 40 (rSV40) is difficult to serve as candidate vector for in vivo gene therapy, due inefficient production and induction of immune neutralization. The study provides a novel sight that rSV40 triggered neutralizing antibody production, inconsistent with previous reports. Most of results are solid and convincing. However, some concerns and more experiments are need to further verify.

1. In vitro, authors used the human liver-derived cell lines (Huh7 and HepG2) to test the activation of hybrid liver specific promoter, they should use mouse liver-derived cell lines to test the promoter activity as well.

**Response:** We agree that in view of the poor efficacy in vivo, showing that the promoter used is active in mouse hepatocytes is relevant. Although we have not tested this promoter in mouse hepatoma cells in vitro, we have used it activity of this promoter has been demonstrated in vivo in mouse liver. In AAV vectors this promoter provides the expression of UGT1A and human UGT1A1 in mouse liver upon systemic injection. Since this is indeed relevant we inserted the references to these previously published studies. The reference 27 and 28.

2. Authors are necessary to detect the expression of hUGT1A1 in mRNA and protein levels in liver from mice injected by rSV40 vectors.

**Response:** Showing mRNA expression is indeed a final prove of the expression from these constructs. We have determined mRNA expression using qPCR in the liver of the treated animals. In contrast to our studies using AAV, the mRNA levels in mice treated with the rSV40 vector were very low. Although a signal was detected in some of the treated mice, the levels were too low for reliable quantification using this very sensitive method, again confirming that rSV40 vectors appear very in-efficient for liver transduction in vivo.

3. In figure 4 and 5, statistical analysis methods should be included in figure legends.

**Response:** We do apologize for this omission and thank the reviewer, and have included the statistical analysis methods in figure 4 and 5.

4. There is no data description the “lack of purification method to produce pure high tittered batches” in title, hence the title needs to be amended.

**Response:** we thank the reviewer for this comment. As mentioned a good production system for rSV40 has been developed using the newly developed super Vero cell line. The generation of high titered vector needed for in vivo experiments depends on highspeed centrifugation. No methods have been developed to ensure the removal of cellular debris. Presence of this, consisting of cell membrane and DNA, can be toxic upon administration to the systemic circulation, explaining the toxicity seen in mice when increasing the dose. We do agree with the reviewer that we did not investigate the toxicity nor did we show presence of cell debris. Therefore we do agree with changing the title: Low efficacy of rSV40 in Ugt1a1^-/-^ mice with severe inherited hyperbilirubinemia.

5. Authors needs to present some data about the Ugt1a gene being knocked out in mice.

**Response:** We thank the reviewer for this comment and have added additional describing the generation of this model and the data obtained in this model in our previous study (Bortolussi et al, 2012, FASEB J.)

Line 119-122: The Ugt1a-/- mice have a one nt deletion in exon 4 of the Ugt1a locus causing a shift in the reading frame introducing a stop codon immediately after the deletion. Due to the absence of the co-substrate binding and transmembrane domains the truncated enzyme is inactive, as published previously (14).

6. English/grammar should be improved for clarity, e.g. in 173-174 line, wass should be corrected to was.

**Response:** we thank the reviewer for helping us and have corrected this mistake.

**Reviewer #2:** This manuscript basically reported negative results. The reviewer appreciated the hard work and the authors’ scientific integrity. It is not clear whether the same viral vectors were used for in vitro and in vivo experiments. The expression of transgene in the liver should be detected by Western blot.

**Response:** we thank the reviewer for your recognition of our work. The viral vectors used in vitro and in vivo were identical. Both rSV-hUGT1A1 and rSV-HLP-hUGT1A1 were produced by AMARNA Therapeutics in Leiden, the Netherlands.

By treating the mutant mice with SV40 vectors, the expression of the transgene was too low to be detected by WB. We have seen in other gene therapy experiments using AAV vectors, that if the efficacy of the treatment is not enough to reduce plasma bilirubin levels below 3-4 mg/dL, the transgenic protein cannot be detected by WB analysis.

Minors,
Line 44, why there is only one sub-title for Introduction?

**Response:** We agree with the reviewer that this one sub-title is strange and have removed it.

Line 204, rSV40 should be rSV.

**Response:** we thank the reviewer’s point out that, and we change rSV40 to rSV in the manuscript.
