# Supplementary material for: Low efficacy of recombinant SV40 in Ugt1a1-/- mice with severe inherited hyperbilirubinemia
Source: PLoS One. 2021 Apr 23;16(4):e0250605. doi: 10.1371/journal.pone.0250605 (PMC8064607; doi:10.1371/journal.pone.0250605)
Supplement: Attachment — Submitted filename: Response to reviewers.docx [file pone.0250605.s005.docx]

Dr. P. J. Bosma

Amsterdam University Medical Centers

Tytgat Institute for Liver Research

Meibergdreef 69

1105 BK Amsterdam

The Netherlands

Dr. Joerg Heber

Editor-in-Chief

PLOS ONE

April 6th 2021

Dear dr. Joerg Heber,

We are grateful for your potential interest in our manuscript entitled manuscript “*Low efficacy of recombinant SV40 in Ugt1a1-/- mice with severe inherited hyperbilirubinemia* (ID: PONE-D-20-33243)”. We thank the reviewers for their comments and suggestions to improve our manuscript. A manuscript with the tracked changes in red and a clean version are submitted. Please find our point-by-point response below.

Yours sincerely,

Xiaoxia Shi

Dr. Piter Bosma

**Reviewers' comments:**

**Reviewer #1:** Authors have complemented the revision. I do not any other questions. If it is allowed，i hope these authors to provide a higher aesthetic picture！

**Response:** We thank the review’s suggestion, next time we will use a more professional tool to make figures and pictures.

**Reviewer #2:** The reviewer is disappointed that the authors decline to perform additional experiments. The reviewer understand that this manuscript basically reported negative results. But to make the story complete, some missing data should be provided. Only citing an almost ten-year old reference (Bortolussi et al, 2012, FASEB J.) is not enough. The reviewer recommends a Western Blot assay showing Ugt1a1 expression in the mice liver. The groups should include WT mice, Ugt1a1-/- mice, rSV40 treated mice, and probably rAAV treated mice from the previous reports. The authors should retain some tissues from the previous research. It is very hard to understand why the authors decline to perform a simple WB assay. The ratio of the rSV40- and rAAV-mediated transgene expression is very important for the reader to make a conclusion.

**Response:** Since using a very sensitive qPCR assay we did show the expression of hUGT1A1 mRNA in the rSV40 treated animals is close to background levels the presence of UGT1A1 protein in liver is expected to be undetectable. We do understand the point raised by the reviewer that a western will further strengthen our message that liver transduction by rSV40 vectors is very inefficient and have therefore performed this experiment. Using an anti-UGT1 rabbit polyclonal antibody (Santa Cruz Biotechnology, Santa Cruz, CA) to detect UGT1A1 expression in liver of Ugt1a1 deficient mice treated with rSV40 and for comparison, treated with rAAV8 and in wild type mice. This western has been added to the paper (Fig 4D) and indeed illustrates the major difference in efficacy of these two vectors in a very clear way. We therefore thank this reviewer for being persistent at this point.

The results of this western are presented as follows:

Line 223-228: The hUGT1A1 expression in liver was determined by western blot showing signals in mice treated with rSV-h*UGT1A1* or rSV-HLP-h*UGT1A1* comparable to that in PBS treated Ugt1a1 deficient mice (Fig 4D). In contrast administration of a 10 fold lower dose of AAV8 vector, containing hUGT1A1 behind the same liver specific promoter (AAV-L), did show presence of hUGT1A1 while treatment with a comparable dose of this vector (AAV-H), showed a very prominent presence of UGT1A1.

This result is also mentioned in the discussion as follows:

Line 268-270: A dose of 1x10^11^ vg/kg results in therapeutic correction of serum bilirubin levels (28) and in contrast to both rSV40 vectors, in clearly detectable levels of UGT1A1 in the liver (Fig 4D).

The legend of Fig 4D reads as follows:

Line 475-478: (D) Presence of UGT1A1 in Ugt1a1 deficient mice treated with 1x10^11^ vg/kg of AAV8-h*UGT1A1* (AAV L), PBS, 1.7x10^12^ vg/kg of rSV-h*UGT1A1*, 1.7x10^12^ vg/kg of rSV-HLP-h*UGT1A1*, or 1x10^12^ vg/kg of AAV8-h*UGT1A1* (AAV H), and in a non-treated wild type mouse (WT). Calnexin was used as loading control on the same membrane.
